# Hospital Meetings

**Published:** 1901-06-08

**Authors:** 


### HOSPITAL MEETINGS.

### MIDDLESEX HOSPITAL.

Sir Ralph Thompson, the chairman of the board, presided at the quarterly Court of Governors of the Middlesex Hospital on May 30th ultimo. The report presented opened with the announcement that his Majesty had signified his consent to continue the Royal patronage of the hospital, thus following the example of her late Majesty and her predecessors William IV. and George IV. In conformity with established precedent, the designation of "Queen" Ward had been altered to "King" Ward. The board reported that the building extensions to the north-east wing were satisfactorily approaching completion. These alterations provide for the enlargement of two of the wards and the conversion of another into kitchen offices, and an entirely new sanitary block for the whole of the enlarged wing. The tender of Messrs. John Grover & Sons for £4,388 had been accepted, with one of £643 from Messrs. John Benham & Sons for the requisite cooking apparatus and fittings. This expenditure, with £700 for the purchase of ground at Hendon for the erection of laundry premises, the meeting was asked to sanction. Plans and estimates for the proposed buildings were now receiving the consideration of the board, who were availing themselves of the advice of Mr. W. Massey, consulting laundry engineer, and it was hoped to commence building operations very shortly. The architect's plans in connection with the extension of the nursing home were submitted for sanction. No scheme for the centralisation of the boiler power had so far been decided on, but the board hoped shortly to put forward satisfactory proposals with regard to it. Sir Ralph Thompson and Sir Arthur Watson had been re-elected chairman and deputy-chairman of the board respectively. Sir Ralph Thompson had retired from the chairmanship of the Council of the Medical School and had nominated Sir Arthur Watson as his successor. Mr. Horace Cockerell had been appointed a member of the Council of the Medical School in the place of Mr. Frank Debenham. Lord Howard de Walden had intimated his intention of subscribing £300 per annum to the hospital funds, and the meeting was asked to confirm his appointment as a vice-president. His Lordship had also consented to distribute the prizes to the medical students at the opening of the schools for the winter session on October 1st. £1,000 had been received from the trustees of the "David Hughes" fund. In the Medical School, the newly-created office of Obstetric Tutor had been filled by the appointment of Dr. Comyns Berkely. Satisfaction was expressed with the working of the convalescent home, which during the quarter ended March 31st last afforded its benefits to 174 patients (men, women, and children) and 7 nurses. In the hospital itself during the quarter 1,166 medical and surgical patients were treated. In the female cancer wing 28 patients had been admitted, and there had been 24 deaths. In the out-patient department 10,750 patients were treated, the total number of attendances being 25,718, giving an average of 2½ attendances for each patient, and an average of 282½ patients seen daily.

The report was adopted, and the customary routine business having been duly attended to the meeting separated.

### HOSPITAL FOR SICK CHILDREN.

Mr. Arthur Lucas, the chairman of the Committee of Management, presided at the forty-ninth annual general Court of Governors of the Hospital for Sick Children, Great Ormond Street, on May 31st ultimo.

After referring in suitable terms to the death of Her late Majesty, the report went on to recapitulate how, in January, 1900, the serious financial position of the hospital was brought to the notice of "Mr. Punch," and an appeal was issued by him, resulting in no less a sum than £16,632 being collected, £576 of which was new annual subscriptions. But for this assistance the resources of the hospital would have been exhausted. Previous appeals to the public had not met with the response the committee had expected; in response to "Mr. Punch," however, donations and subscriptions flowed in from all parts of the world. With the money so provided the debt of £4,500 to the bankers was paid off and sufficient funds provided for the immediate needs of the hospital. Naturally the warmest gratitude was expressed to all who assisted in the matter. During the year the hospital was partially closed for seven weeks—from May 9th to June 6th, and from July 7th to July 28th—and entirely closed for five weeks—from June 6th to July 7th. This was rendered necessary by the water cisterns being worn out and the need of a new main, owing to the growth of the hospital; and the opportunity was taken to remodel the drainage system. The closing of the wards necessarily affected the work of the hospital, reducing the number of patients treated, but increasing the cost per head as compared with previous years. The new in-patients numbered 1,690, a decrease of 272 as against 1899. The new out-patients numbered 19,837, a decrease of 4,055. The out-patients' attendances numbered 79,061, as compared with 94,156 in 1899. The number of patients sent to Cromwell House Convalescent Home was 154, a decrease of 59. The cost of each in-patient per week was in 1900 £1 15s., and in 1899 £1 10s. 11¼d. The out-patients cost in 1900 2s. 8½d., and in 1899 1s. 10¾d. The ordinary income for the year was £11,505, and the ordinary expenditure £15,758. The extraordinary income, including the "Punch" appeal donations, was £20,490, of which £2,484 was legacies, this last figure showing a great falling-off from the average. The extraordinary expenditure amounted to £3,373, and comprised, among other items, sums for repairs, building improvements, water drainage, and £865 for the reconstruction of the in-patient operating theatre. A fourth donation was acknowledged from the Prince of Wales's Fund of £500, to enable the committee to open the Goldsmiths' Ward for the treatment of whooping cough. Sixteen beds were opened in October, and up to December 31st 40 patients had been admitted. To keep this ward going would cost £1,000 a year, and a special appeal was made for new annual subscriptions to meet this charge. £24,000 had been raised on mortgage at 3¼ per cent. to pay off the balance of £20,000 due to the Trustees of the Roman Catholic Hospital, from whom the adjoining property was purchased in 1898 for £30,000, and to repay £3,000 to the hospital on account of the sums expended in fitting and adapting the nurses' house. The fees from probationer nurses were devoted to the payment of the interest. The nurses supplied for private cases were in growing demand with the public, there being now a staff of 27 in constant work. Warm thanks were expressed to the Working Guild which supplies the clothes for the children in the hospital. During 1900 about 1,000 garments were provided. In recognition of his assistance in connection with the "Punch" appeal, Sir Wm. Agnew had been elected a vice-president. Two vice-presidents had died during the year—Lord Gort, whose connection with the institution extended over 43 years; and Sir James Paget, who for 20 years had laboured to promote the efficiency of the work done. From the Committee of Management Sir John Wolfe Barry had, by his many engagements, been obliged to resign his seat. Mr. John H. Morgan, senior surgeon, had resigned his post, the vacancy being filled by the election of Mr. Chas. A. Ballance. Mr. Morgan, who had served the hospital for 22 years, had accepted the post of Consulting Surgeon. A fourth physician being required to meet the extra work from the additional ward, Dr. Voelcker had been elected, and his place as Assistant Physician had been filled by the election of Dr. F. J. Poynton. Dr. Lee Dickinson had resigned his post as Assistant Physician, and Dr. Robert Hutchinson had been elected to fill the vacancy. Regret was expressed that Dr. Dickinson should have found his health unequal to the strain involved in working for the Children's and for St. George's Hospital. His distinguished father had done so much for them that the committee looked forward to the career of the son being also associated with Great Ormond Street. Mr. Francis J. Steward had been elected as Assistant Surgeon in place of Mr. Ballance. Mr. Donald Gunn had, owing to ill-health, resigned his post as Ophthalmic Surgeon after eight years of good service. Mr. W. T. Lister had succeeded him. Mr. Fairbank had been elected Medical Superintendent on the resignation of Mr. R. G. Ralston. In concluding their report, the committee laid emphasis on the fact that they were facing a prospective deficit of £8,000 per annum, and made a special appeal for funds to meet it.

The CHAIRMAN moved the adoption of the report. Beyond what was mentioned in it, he said they had had no events. They had been quietly but hard at work. The enormous number of cases treated, he thought, reflected the greatest credit on their medical and nursing staff, especially as that work had been done without a single complaint with regard to it reaching the committee. He then touched upon the various points referred to in the report, explaining and justifying what had been done. They were not extravagant he maintained; but their expenses seemed to increase by leaps and bounds. This was largely due to the greater demands made by modern requirements. The cost of operations was far greater than it used to be, while research in medical science involved ever-increasing expense. But the knowledge obtained benefited both rich and poor, and if the public but knew how many of the lives of their dear ones were saved as a result of their investigations, they would not have to go cap in hand for the wherewithal to carry on the work.

The report was unanimously adopted, and Lord ABERDARE having moved a vote of thanks to the King and Queen for granting their patronage, and the retiring officers having been re-elected, the meeting terminated with a very cordial vote of thanks to the Chairman.

